# Ampelopsin Suppresses Stem Cell Properties Accompanied by Attenuation of Oxidative Phosphorylation in Chemo- and Radio-Resistant MDA-MB-231 Breast Cancer Cells

**DOI:** 10.3390/ph14080794

**Published:** 2021-08-12

**Authors:** Vi Nguyen-Phuong Truong, Yen Thi-Kim Nguyen, Somi-Kim Cho

**Affiliations:** 1 Interdisciplinary Graduate Program in Advanced Convergence Technology and Science, Jeju National University, Jeju 63243, Korea; phuongvi.truongnguyen@gmail.com (V.N.-P.T.); ntkyen.hcmus@gmail.com (Y.T.-K.N.); 2 Subtropical/Tropical Organism Gene Bank, Jeju National University, Jeju 63243, Korea

**Keywords:** ampelopsin, medicinal plants, breast cancer, NF-κB signaling, cancer stem cells, resistance, oxidative phosphorylation, mitochondrial respiration

## Abstract

Ampelopsin, also known as dihydromyricetin, is a commonly found flavonoid in medicinal plants. The cancer stem cell (CSC) population is a promising target for triple-negative breast cancer (TNBC). In this study, flavonoid screening was performed in the established MDA-MB-231/IR cell line, which is enriched in CSCs. Ampelopsin suppressed the proliferation and colony formation of stem cell-rich MDA-MB-231/IR, while inducing their apoptosis. Importantly, ampelopsin displayed an inhibitory impact on the stemness features of MDA-MB-231/IR cells, demonstrated by decreases in mammosphere formation, the CD44^+^/CD24^−/low^ population, aldehyde dehydrogenase activity, and the levels of stem cell markers (e.g., CD44, MRP1, β-catenin, and KLF4). Ampelopsin also suppressed the epithelial–mesenchymal transition, as evidenced by decreases in migration, invasion capacity, and mesenchymal markers, as well as an increase in the epithelial marker E-cadherin. Moreover, ampelopsin significantly impaired oxidative phosphorylation by reducing the oxygen consumption rate and adenosine triphosphate production in MDA-MB-231/IR cells. Notably, ampelopsin treatment significantly reduced the levels of the phosphorylated forms of IκBα and NF-κB p65, as well as the levels of tumor necrosis factor (TNF)-α-stimulated phosphorylation of IκBα and NF-κB p65. These results demonstrated that ampelopsin prevents the TNF-α/NF-κB signaling axis in breast CSCs.

## 1. Introduction

Breast cancer is the most popular disease and driving cause of cancer death in women. According to the Global Cancer Statistics 2020 produced by the International Agency for Research on Cancer, breast cancer comprised 24.5% of cancers diagnosed in women, representing the highest incidence in most countries worldwide [1]. Triple-negative breast cancer (TNBC) is an aggressive sub-group of breast cancer that lacks the expression of progesterone receptor (PR), estrogen receptor (ER), and human epidermal growth factor receptor 2 (HER2). TNBC is characterized by resistance to therapy, high rates of metastasis, and recurrence. Thus, there is an urgent necessity to develop novel treatment strategies for TNBC patients, especially for patients with distant metastases [2].

Cancer stem cells (CSCs) comprise tumor-initiating cells in heterogeneous tumors. The presence of CSCs in breast tumors is a leading cause of failed current breast cancer treatments [3]. Many previous studies have reported the specific signatures of breast cancer stem cells (BCSCs), such as CD44^+^/CD24^−/low^, aldehyde dehydrogenase^high^ (ALDH^high^), or CD133^+^ genotypes [4]. Several pluripotent transcription factors, including OCT4, Sox2, Nanog, MYC, and Krüppel-like factor 4 (KLF4), are associated with the biological activities of CSCs. In addition, components of major intracellular signaling pathways (e.g., Notch, Wnt, Hedgehog, JAK-STAT, PI3K/AKT/mTOR, and nuclear factor-κB [NF-κB]) are enriched in CSCs and are important regulators of their characteristics [3,5]. In previous research, we established TNBC MDA-MB-231/IR cells, which showed greater stemness features (e.g., migration, invasion, chemoresistance, and radioresistance) compared with parental MDA-MB-231 cells. Furthermore, transcriptomic analysis demonstrated the upregulation of NF-κB signaling, tumor necrosis factor (TNF-α), and Toll-like receptors in these cells [6,7].

NF-κB, a crucial transcription factor frequently observed in breast cancer, can regulate several genes involved in cell survival, proliferation, cellular transformation, oncogenesis, and metastasis [8,9]. In the resting state, the NF-κB dimer is existent in an inactive complex with its inhibitor (IκB) in the cytoplasm, which prevents nuclear import. Activation of NF-κB signaling occurs through two main pathways: the canonical and non-canonical pathways. According to the canonical pathway, several stimuli (e.g., lipopolysaccharide, TNF-α, and interleukin-1) activate the inhibitor of κB kinase (IKK) complex, resulting in IκB phosphorylation, ubiquitination, and proteasomal degradation. Loss of IκBα enhances the translocation of NF-κB to the nucleus, stimulating the transcription of its gene targets. The NF-κB family comprises five members: p65 (RELA), RELB, cREL, p105/p50, and p100/p52. The amino-terminal Rel homology domain, which has nuclear localization, DNA binding, and homo- and heterodimerization activities, is common among these members. p65 is the most abundant form of NF-κB, which is involved in cancer pathogenesis and is thus an important target for preventive and therapeutic strategies [8,9,10]. Moreover, the overexpression of p65 decreases celecoxib-induced cell death in breast cancer cells [11]. Multiple post-translational modifications of nuclear p65 support its transcriptional activity; in particular, phosphorylation at serine residues 536 and 276 is critical for enhancement of p65 transcriptional activity [12,13]. In addition, the IKKα-mediated induction of p65 phosphorylation at serine 536 is involved in the invasive oncogenic phenotype of Her2^+^ breast cancer cells [14].

Metabolic reprogramming has been reported as a hallmark of CSCs [15]. CSCs respond differently to metabolic adaptations compared with cancer cells. Cancer cells rely on glycolysis for lactate production to adapt to the increased energy demands for proliferation and survival, regardless of the presence of oxygen; this is also known as aerobic glycolysis or the Warburg effect [16]. However, CSCs promote tumor formation by showing a flexible metabolic imprint capable of switching between glycolysis and oxidative phosphorylation (OXPHOS) in mitochondria [17]. In addition, there is increasing evidence to support enhancement of the OXPHOS metabolic phenotype in CSCs to maintain their stemness and metastatic potential, while increasing their resistance to DNA damage [18,19]. Moreover, OXPHOS is reportedly stimulated by an NF-κB-dependent metabolic pathway to support the growth of cancer cells [20]. Therefore, the inhibition of OXPHOS has become a potential option for cancer therapy [21].

Studies have identified a variety of phytochemicals derived from medicinal plants as potential BCSC-targeted therapeutics based on their inhibitory activity on mammosphere formation [22,23] and sensitivity to chemotherapeutic drugs [24]. Recent discoveries have demonstrated that BCSCs rely more on OXPHOS rather than their differentiated counterparts [25]. Therefore, it has become increasingly important to find phytochemicals that can target metabolic phenotypes in BCSCs. In addition, several phytochemicals derived from medicinal plants species have been known to have potential anticancer properties [26]. Accordingly, screening was performed to find substances capable of targeting MDA-MB-231-IR cells, which exhibited greater degrees of chemo- and radio-resistance, in comparison with MDA-MB-231 cells [6,7], among various phytochemicals reported to have anticancer efficacy against breast cancer [27,28,29,30,31,32]. Ampelopsin, also known as dihydromyricetin, is one of the primary flavonoids in medicinal plants such as Japanese raisin tree (*Hovenia dulcis* Thunb.) and Chinese Rattan tea (*Ampelopsis grossedentata)* [33,34,35]. Ampelopsin can effectively promote apoptosis and suppress proliferation and metastasis in different cancers by modulating various signaling pathways such as ERK1/2, JNK pathway, PI3K/AKT, and mTOR signaling [36]. Moreover, ampelopsin markedly promoted the sensitivity to paclitaxel and doxorubicin in resistant ovarian cancer cells [37]. Further, ampelopsin has shown potential as a therapeutic agent in multiple metabolic diseases [35,38]. However, little is known regarding its effects on CSCs, and in particular, the effect of ampelopsin on the metabolism in BCSCs has not been reported yet. In this study, we examined the therapeutic potential and ability of ampelopsin to modulate the glucose metabolism in TNBC MDA-MB-231/IR cells with stemness properties.

## 2. Results

### 2.1. Ampelopsin Promotes Cytotoxicity in MDA-MB-231/IR Cells

**Figure 1:**
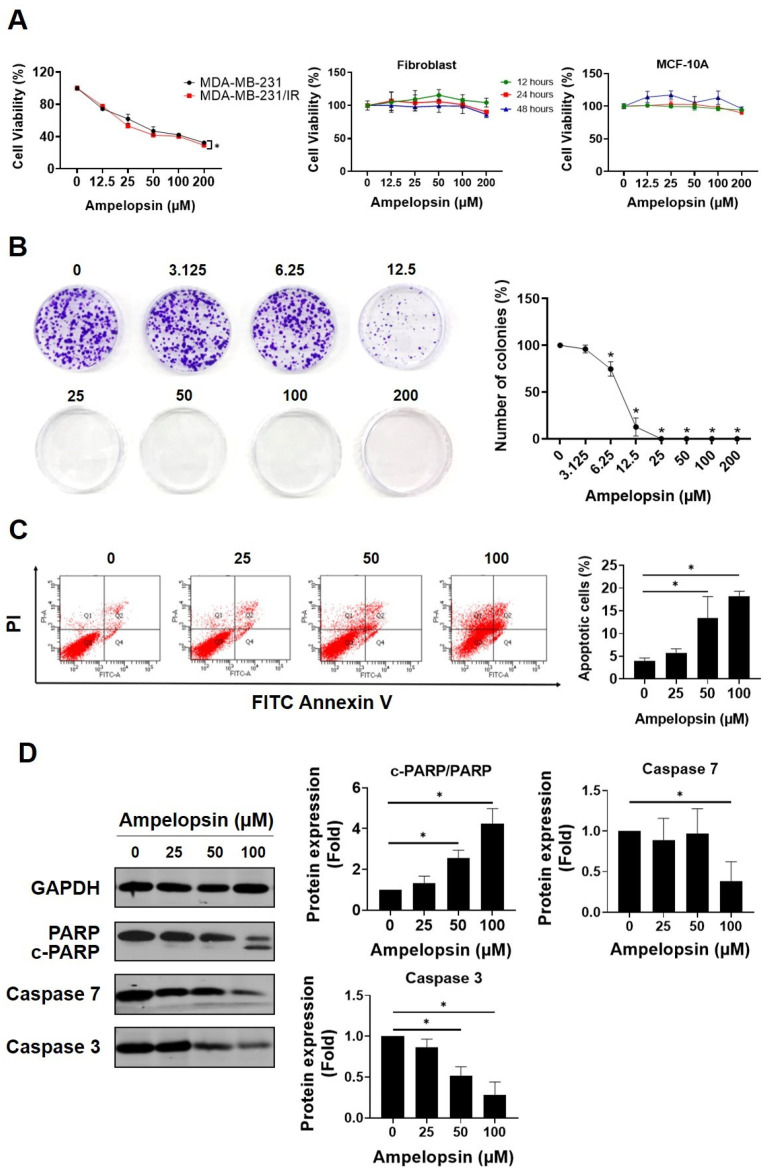
Ampelopsin exerts cytotoxicity in MDA-MB-231/IR cells. (**A**) The cell viability was measured by MTT assay after ampelopsin exposure for 24 h in MDA-MB-231 and MDA-MB-231/IR cells or fibroblast cells and the mammary epithelial cells, MCF-10A, in dose- and time-dependent manners. (**B**) Colony formation assay of cells treated with ampelopsin after 10 days of incubation. (**C**) Annexin V vs. propidium iodide (PI) staining was conducted to measure the apoptotic population by FACS analysis, following treatment with ampelopsin for 24 h. (**D**) Western blotting analysis of apoptosis markers following ampelopsin treatment. GAPDH was used as the loading control. Original films were shown in Figure S3. * p < 0.05 vs. control.

The MTT assay was performed to screen several well-known cancer-targeting flavonoids in both MDA-MB-231 cells and resistant MDA-MB-231/IR cells. Among tested phytochemicals, ampelopsin had chemotherapeutic potential against MDA-MB-231/IR cells (Figure S1). Compared with parental cells, ampelopsin had a greater cytotoxic impact on MDA-MB-231/IR cells (IC_50_ 58 ± 3.84 µM vs. IC_50_ 42.89 ± 1.86 µM) after 24 h of exposure (Figure 1A). Therefore, ampelopsin was selected for further experiment on resistant MDA-MB-231/IR cells. Furthermore, ampelopsin had no cytotoxicity toward normal fibroblast cells or MCF-10A cells (Figure 1A), indicating that ampelopsin was nontoxic on normal human cell lines. Using the doses lower and higher than IC_50_, treatment with ampelopsin significantly reduced colony formation by MDA-MB-231/IR cells to approximately 75% and 12.8% ± 4.7% of control at doses of 6.25 μM and 12.5 μM, respectively, and no colonies at doses of 25, 50, 100, and 200 μM after 10 days of exposure (Figure 1B). In addition, we checked whether ampelopsin could induce apoptosis in resistant cells. Notably, ampelopsin increased the proportion of apoptotic cells by 18.2% ± 1.08% at a dose of 100 μM, as shown by annexin V/propidium iodide staining (Figure 1C). Furthermore, ampelopsin-treated MDA-MB-231/IR cells displayed a decrease in the expression of caspase 3 by 0.28-fold and caspase 7 by 0.38-fold. In addition, one of important substrates of caspase cascade, the cleavage of poly(ADP-ribose) polymerase (PARP) from the 116 kDa form to the 89 kDa protein, was reportedly required for apoptosis in several cell lines [39,40]. In non-small cell lung cancer (NSCLC), the cancer stem cell population showed less apoptotic response after cisplatin treatment, as demonstrated by diminished cleavage of PARP from full-length fragment and caspase 3 activation [41]. In this study, ampelopsin significantly decreased the level of full-length PARP and enhanced cleaved fragment, displayed by an increase in the ratio of cleaved-PARP/PARP by 4.24-fold at a dose of 100 μM, compared with untreated control (Figure 1D).

### 2.2. Ampelopsin Treatment Attenuates Cancer Stem Cell Features in MDA-MB-231/IR Cells

**Figure 2:**
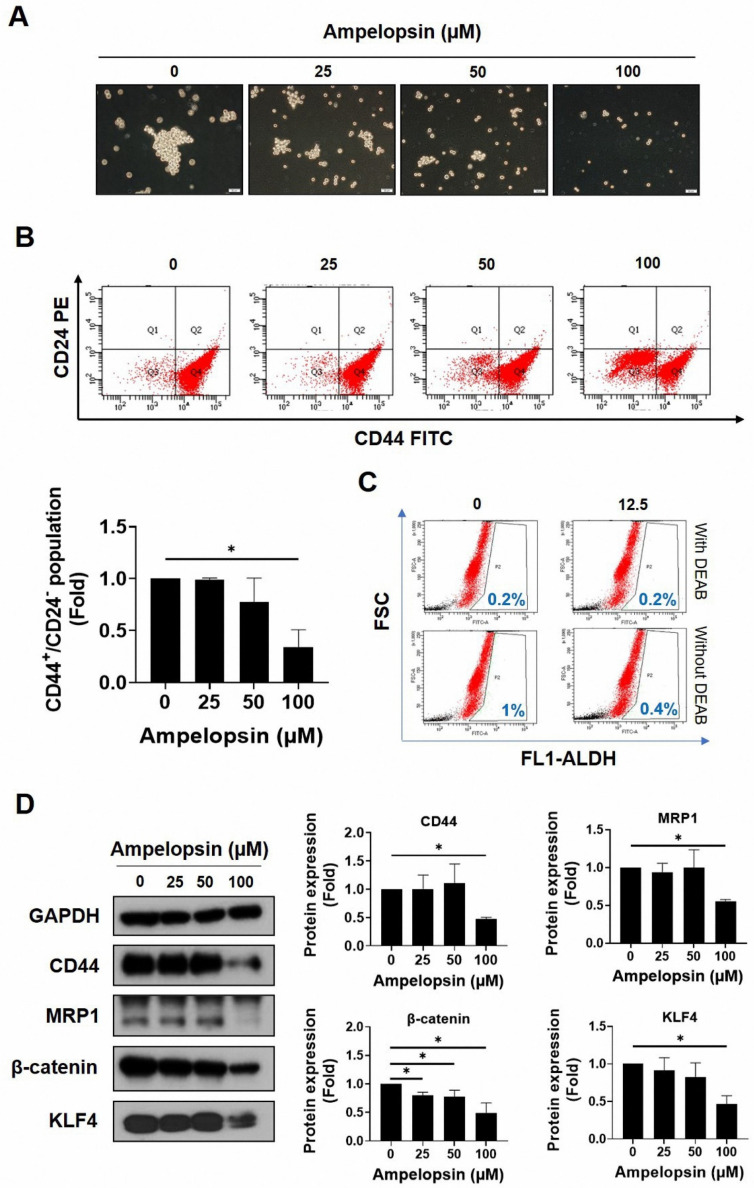
The suppression of ampelopsin on stem-like cell characteristics in MDA-MB-231/IR cells. (**A**) Effect of ampelopsin on mammosphere formation after 10 days of treatment (100× magnification). (**B**) The CD44^+^/CD24^−/low^ population was measured by FACS after 24 h treatment of ampelopsin. (**C**) ALDH^+^ population after ampelopsin treatment was detected using Aldefluor assay kit and 4-diethylaminobenzaldehyde was considered as a negative control. (**D**) Western blotting analysis of stemness markers after incubation 24 h with ampelopsin. GAPDH was used as the loading control. Original films were shown in Figure S4. * p < 0.05 vs. control.

As MDA-MB-231/IR cells reportedly exhibit more greater stem cell characteristics than do parental cells, we examined whether ampelopsin could inhibit the stemness features of MDA-MB-231/IR cells. The ability to form spheres is associated with the self-renewal ability of CSCs, indicating elevated tumorigenicity and metastaticity [42]. As shown in Figure 2A, ampelopsin sharply reduced the mammosphere formation ability after 10 days of treatment (0, 25, 50, and 100 μM). Moreover, the CD44^+^/CD24^−/low^ population, a well-known subpopulation of breast CSCs, decreased dose dependently following ampelopsin exposure, as shown by FACS analysis (Figure 2B). In addition, ampelopsin reduced the ALDH-positive population to 0.4% (2.5-fold) at the non-lethal concentration of 12.5 μM (Figure 2C). Remarkably, Western blot analysis showed that 100 µM ampelopsin reduced the levels of well-known CSC markers, such as CD44 (0.48-fold), MRP1 (0.55-fold), β-catenin (0.49-fold), and KLF4 (0.47-fold) (Figure 2D) in a dose-dependent manner. These observations indicated that ampelopsin effectively reduced the stemness properties in MDA-MB-231/IR cells.

### 2.3. Ampelopsin Treatment Reduces Invasion and Migration by MDA-MB-231/IR Cells

**Figure 3:**
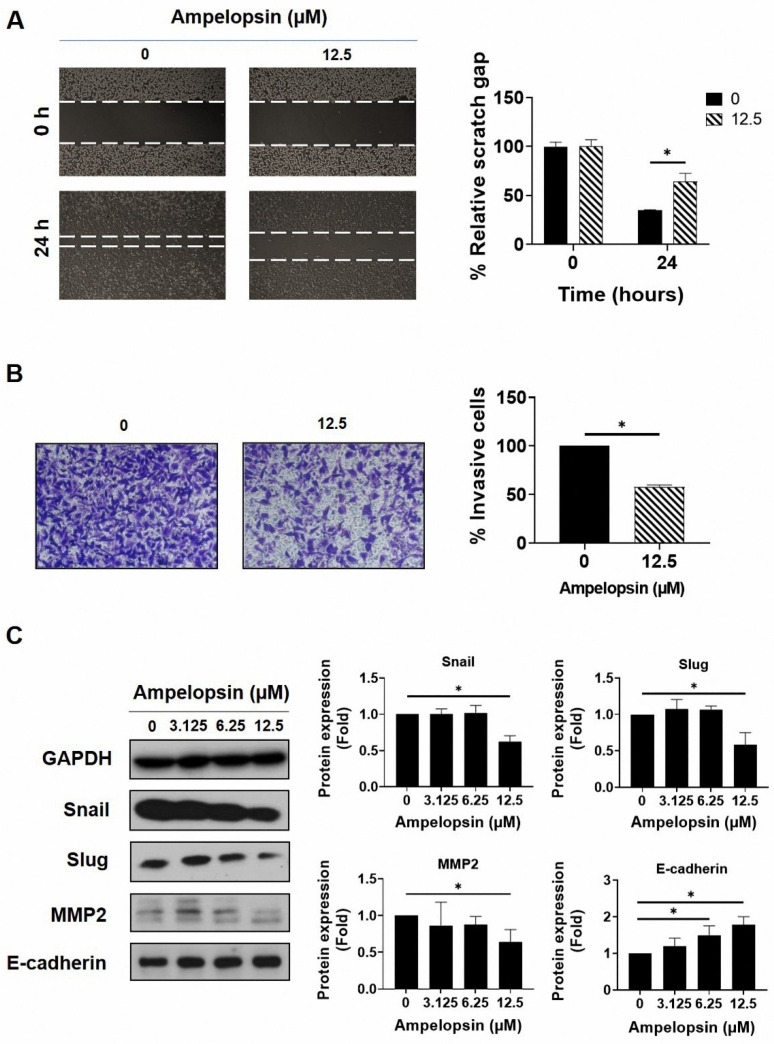
The inhibitory potential of ampelopsin on invasion and migration by MDA-MB-231/IR cells. (**A**) The cell invasion assay was used to measure the invasive cells following treatment 24 h with ampelopsin (12.5 µM). Images were obtained by phase-contrast microscopy (100× magnification). (**B**) Migrated cells after 24 h treatment with ampelopsin was determined by wound healing assay. Images were obtained by phase-contrast microscopy (100× magnification). (**C**) Western blotting analysis was performed for epithelial–mesenchymal transition (EMT) markers following treatment with ampelopsin for 24 h. GAPDH was used as the loading control. Original films were shown in Figure S5. * p < 0.05 vs. control.

The epithelial–mesenchymal transition (EMT) is an important stem cell property involved in metastasis and resistance to therapy [43]. Therefore, we examined the capacity of ampelopsin to suppress invasion and migration by MDA-MB-231/IR cells. Wound healing assay showed that ampelopsin prevented migrated cells at the non-lethal concentration of 12.5 µM after 24 h exposure (Figure 3A). In addition, the cell invasion assay using the Transwell plates demonstrated a decline of invasive capacity by 57.7% ± 0.47% in MDA-MB-231/IR cells incubated with 12.5 µM ampelopsin (Figure 3B). Subsequently, Western blotting was conducted to evaluate the influence of ampelopsin on EMT markers. Accordingly, ampelopsin rapidly reduced the levels of the mesenchymal markers, Snail (0.62-fold), Slug (0.59-fold), and MMP2 (0.64-fold) at non-lethal concentration. Furthermore, ampelopsin markedly enhanced the level of the epithelial marker, E-cadherin (Figure 3C). All these data demonstrated that ampelopsin inhibited invasive and migratory capacity by MDA-MB-231/IR cells.

### 2.4. Ampelopsin Impairs OXPHOS in MDA-MB-231/IR Cells

**Figure 4:**
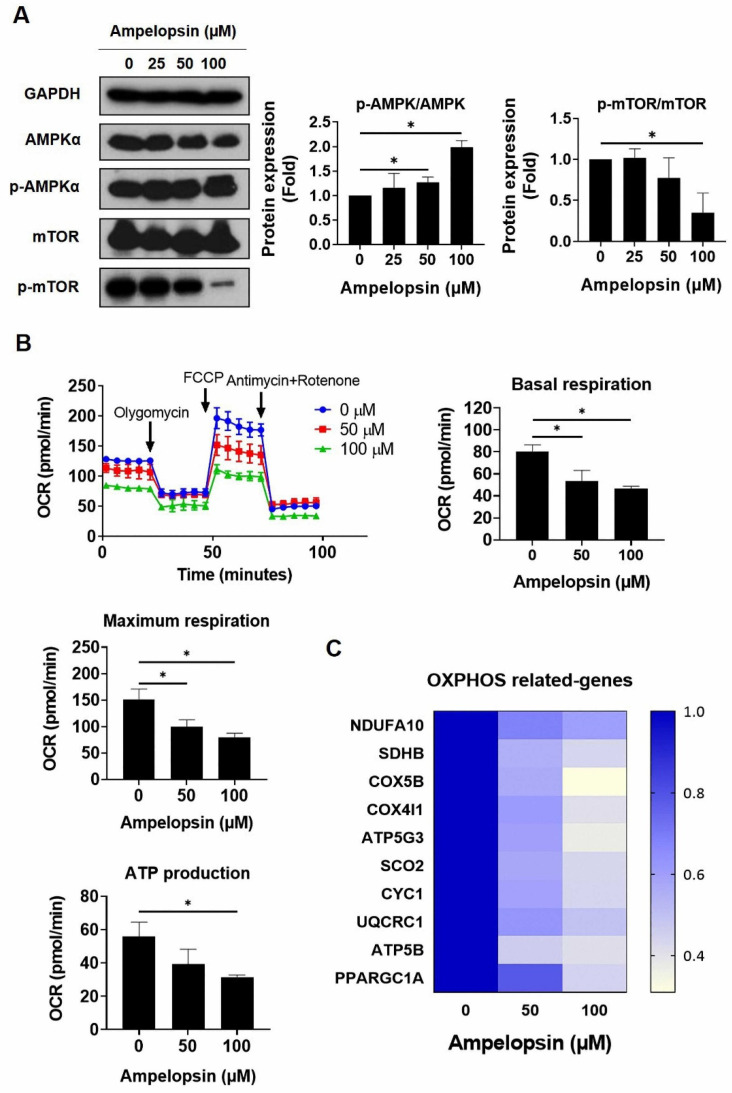
Assessment of oxidative phosphorylation in MDA-MB-231/IR cells after ampelopsin treatment. (**A**) Levels of AMPKα, p-AMPKα, mTOR, and p-mTOR were examined by Western blotting analysis after 24 h incubation with ampelopsin. Original films were shown in Figure S6. (**B**) Seahorse XF analysis was performed to measure OCR, ATP production, basal respiration, and maximum respiration after ampelopsin treatment for 24 h. Cells were cultured in XF assay medium supplemented with pyruvate (1 mM), glucose (10 mM), and l-glutamine (2 mM), and then treated with oligomycin (2 µM), carbonyl cyanide-*p* trifluoromethoxyphenylhydrazone (1 µM), antimycin (1 µM), and rotenone (1 μM). (**C**) Real-time PCR analyzed the expression of OXPHOS-related genes. * p < 0.05 vs. control.

Several studies have reported that CSCs rely on mitochondrial metabolism to promote tumorigenesis, thus the inhibition of OXPHOS in cancer cells can represent a new therapeutic target [21]. Ampelopsin has been reported to control the signaling pathways associated with glucose metabolism and improved the cellular damage caused by toxic materials [38]. However, the effects of ampelopsin on metabolism in cancer are not fully understood. Notably, MDA-MB-231/IR cells were noted to have a 0.56-fold reduced phosphorylation of adenosine monophosphate (AMP)-activated protein kinase (AMPK) and a 1.5-fold increased level of phosphorylated mTOR, in comparison with parental cells (Figure S2; original films were shown in Figure S10). Interestingly, ampelopsin treatment increased the phosphorylation of AMPK by 2.12-fold, while it decreased the phosphorylation of mTOR by 0.52-fold, compared with untreated controls in resistant cells (Figure 4A). Because of the effect of ampelopsin on AMPK activation, we examined whether ampelopsin could target glucose metabolism in MDA-MB-231/IR cells. XF Seahorse analysis demonstrated that ampelopsin reduced the oxygen consumption rate (OCR). Treatment with ampelopsin also decreased the levels of basal respiration, maximum respiration, and adenosine triphosphate (ATP) production, reaching 46.4% ± 2.31%, 79.2% ± 7.99%, and 31.6% ± 1.15%, respectively; 100 μM ampelopsin caused impairment of mitochondria respiration (Figure 4B). Accordingly, the expression of OXPHOS-related genes was decreased after ampelopsin treatment, represented by real-time PCR experiment: NADH/ubiquinone oxidoreductase subunit A10 (*NDUFA10*), succinate dehydrogenase complex subunit B (*SDHB*), cytochrome c oxidase subunit 5B (*COX5B*), cytochrome C oxidase subunit 4 isoform 1 (*COX4I1*), ATP synthase membrane subunit C3 (*ATP5G3*), synthesis of cytochrome C oxidase 2 (*SCO2*), cytochrome C1 (*CYC1*), ubiquinol-cytochrome C reductase core protein 1 (*UQCRC1*), ATP synthase F1 subunit beta (*ATP5B*), and peroxisome proliferator-activated receptor-gamma coactivator 1-alpha (*PPARGC1A*) (Figure 4C). These data suggested that ampelopsin treatment can suppress OXPHOS in MDA-MB-231/IR cells.

### 2.5. Ampelopsin Suppresses NF-κB Activity in MDA-MB-231/IR Cells

**Figure 5:**
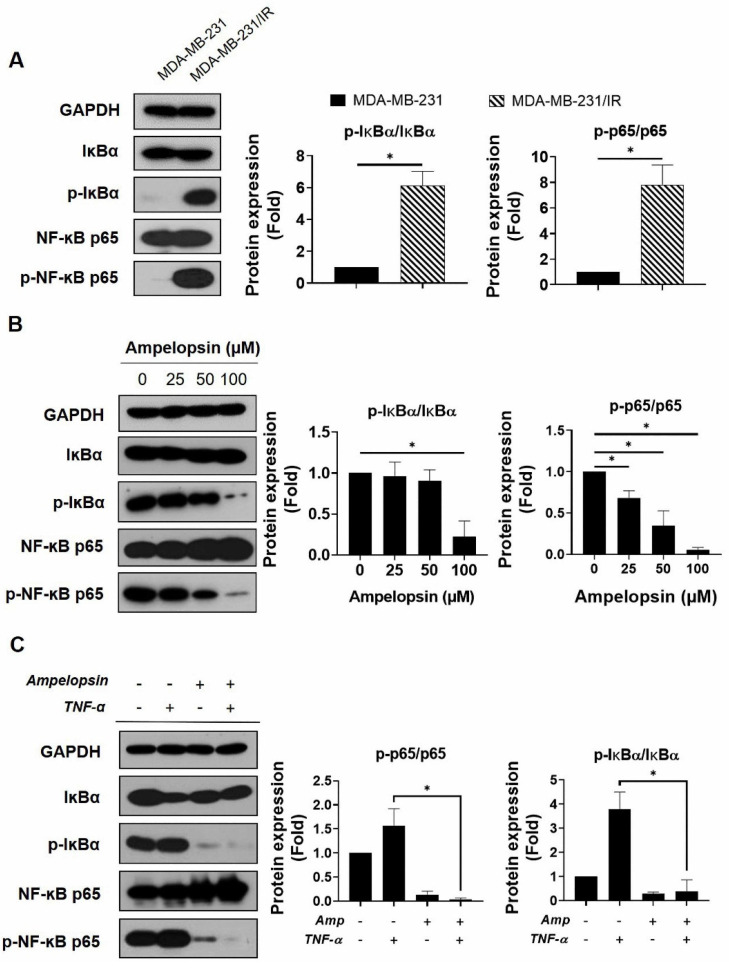
NF-κB inhibition by ampelopsin was determined in MDA-MB-231/IR cells. Representative results of Western blotting indicating (**A**) the levels of IκBα, p-IκBα, NF-κB p65, and p-NF-κB p65 in MDA-MB-231 and MDA-MB-231/IR cells. Original films were shown in Figure S7; (**B**) the levels of IκBα, p-IκBα, NF-κB p65, and p-NF-κB p65 in MDA-MB-231/IR cells after 24 h treatment with ampelopsin. Original films were shown in Figure S8; and (**C**) the effects of ampelopsin on the TNF-α-stimulated NF-κB activity. In this experiment, cells were pretreated with 100 µM ampelopsin before exposure to 10 ng/mL TNF-α for 12 h. Original films were shown in Figure S9. GAPDH was used as the loading control. * p < 0.05 vs. control.

The activation of transcription factor NF-κB regulates several target genes involved in cancer progression and therapy resistance [9]. In addition, p65 reportedly acts as a central controller of energy homeostasis and metabolic alteration by promoting mitochondrial respiration in cancer cells [20]. Because of the crucial role of NF-κB activation in tumor development and the NF-κB signaling enrichment in MDA-MB-231/IR cells, demonstrated by the transcriptomic data from previous studies [6], we examined whether ampelopsin could suppress NF-κB signaling in these cells. As shown in Figure 5A, the levels of p-IκBα/IκBα and p-NF-κB p65/NF-κB p65 were considerably increased by 6.13-fold and 7.82-fold, respectively, in comparison with parental cells after ampelopsin exposure. Marked decreases in the levels of p-IκBα/IκBα and p-NF-κB p65/NF-κB p65 (0.22-fold and 0.05-fold, respectively) were observed after ampelopsin treatment, compared with untreated controls (Figure 5B). We also examined whether ampelopsin could suppress TNF-α-stimulated NF-κB signaling, because the TNF-α/NF-κB pathway has been known to enhance migration, invasion, and tumorigenesis in metastatic cancer [44]. As shown in Figure 5C, TNF-α significantly increased the levels of p-IκBα/IκBα and p-NF-κB p65/NF-κB p65 (3.88-fold and 1.57-fold, respectively), while simultaneously decreasing the level of IκB (by 0.4-fold) with ampelopsin treatment. Taken together, ampelopsin could suppress TNF-α-stimulated NF-κB signaling, supporting a new function for ampelopsin as an inhibitor of the TNF-α/NF-κB signaling axis in BCSCs.

## 3. Discussion

CSCs play critical roles in the proliferation, drug resistance, and metastasis of tumors; importantly, targeting CSCs in cancer treatment is a challenge [3]. TNBC shows heterogeneity, biological aggressiveness, and specific CSC signatures that lead to therapy resistance, metastasis, and recurrence. Therefore, TNBC shows worse treatment outcomes compared with other breast cancer subtypes [3]. In recent studies, we isolated and evaluated a unique chemo- and radio-resistant TNBC cell line derived from the MDA-MB-231 cells, which we designated as MDA-MB-231/IR cells. These cells showed increased stemness characteristics and therapy resistance compared with the parental cells [6,7]. Therefore, these cells could be suitable for use in screening for phytochemicals as lead compounds for novel targeted therapies for TNBC. In Figure S1, after screening of many flavonoids with putative anticancer activities, we found that ampelopsin had a stronger cytotoxic effect on MDA-MB-231/IR cells rather than on the parental cells. Ampelopsin has been shown to trigger apoptosis and autophagy in several cancer cell lines [37,45,46]; to the best of our knowledge, this is the first study to show that ampelopsin can promote apoptosis in this cell line and may have potential as a therapeutic phytochemical for TNBC with CSC characteristics. Indeed, ampelopsin treatment eliminated the CD44^+^/CD24^−/low^ population, while reducing ALDH activity, the level of KLF4 (an oncogene product responsible for CSC [47]), and the level of β-catenin (a transcription factor for several target CSC genes [48]) (Figure 2).

The activation of AMPK, an important master regulator of cellular energy balance, can regulate metabolic energy and promote ATP production. In addition, current studies suggested that AMPK activation may be a promising target for cancer treatment. Moreover, AMPK activation prevented the translocation of NF-κB from the cytosol to the nucleus in activated macrophages within inflamed skin tissues [49]. The well-known downstream target of AMPK, mTOR, can regulate cell growth, cell survival, and protein synthesis [50]. In non-small cell lung cancer, the LKB1/AMPK axis suppresses mTOR activity and promotes cell growth inhibition [51]. Although several studies have reported the introduction of ampelopsin for the treatment of various metabolic diseases and shown that it can activate AMPK [35,52], its effects in BCSCs were not previously reported. In this study, we found that ampelopsin treatment promoted AMPK activation and suppressed mTOR phosphorylation (Figure 4A). These results suggested that ampelopsin could have a metabolic regulatory function in the targeting of CSC populations in MDA-MB-231/IR cells, prompting us to conduct further experiments to determine how ampelopsin may affect metabolism.

There is increasing evidence that CSCs with high metastatic potential often show enhanced OXPHOS and elevated ATP levels, which aid their separation from the basement membrane in tumors to establish metastasis. Moreover, enhanced mitochondrial oxidative metabolism is crucial for maintaining the CSCs’ properties including metastatic capacity, drug resistance, and stemness characteristic [19]. Therefore, mitochondrial metabolism is an emerging target for elimination of CSCs [21]. Based on these reports, we investigated whether ampelopsin could have a metabolic regulatory role in targeting the stem cell populations in MDA-MB-231/IR cells.

OCR was measured to assess mitochondrial OXPHOS and reflect mitochondrial function [53]. A previous study showed that the HIF1-α inhibitor, LW1564, inhibited mitochondrial respiration by reducing OCR and electron transport chain complex I, which decreased ATP production and promoted the degradation of HIF-1α, thus inhibiting the growth of hepatocellular carcinoma cells. The reduction of total ATP caused an increase in the AMP/ATP ratio, activating AMPK signaling and preventing lipid synthesis in these cells [54]. Notably, reductions of OCR, maximum respiration, basal respiration, and ATP production were observed in MDA-MB-231/IR cells after ampelopsin treatment (Figure 4B), reflecting the key role of ampelopsin in preferentially suppressing the CSC phenotype, which is dependent on mitochondrial respiration for survival. Moreover, *PPARGC1A*, the gene encoding PGC1-α protein, a regulator of OXPHOS metabolism, was significantly overexpressed in circulating tumor cells, and the suppression of PGC1-α markedly weakened the stemness features of CSCs [18,19]. In this study, ampelopsin was found to reduce the level of *PPARGC1A* gene expression (Figure 4C), enhancing the inhibitory effects of ampelopsin on mitochondrial function and ATP synthesis, thus suppressing the growth of MDA-MB-231/IR cells.

NF-κB is a key transcription factor driving the expression of various genes involved in anti-apoptosis, tumor initiation, recurrence, and metastasis in cancer and CSCs [8,9]. The phosphorylation of NF-κB was reported to have an important function in the regulation of NF-κB signaling in many types of cancer [55]. In particular, phosphorylation of the p65 subunit has a profound effect on the enhanced transcriptional activity of NF-κB and control of NF-κB-directed transactivation [13,56]. MDA-MB-231/IR cells with CSC characteristics, which were established in this study, had higher levels of IκB and NF-κB p65 phosphorylation, compared with MDA-MB-231 cells (Figure 5A). This suggested a therapeutic benefit to selectively targeting NF-κB signaling in breast CSCs. Recent reports have revealed a critical role for NF-κB in regulation of cancer metabolic reprogramming. NF-κB was also identified as a regulator of oncogenic mitochondrial functions; p65 upregulated mitochondria respiration by promoting the expression of mitochondrial *SCO2* [20,57]. In this study, the expression of *SCO2* was decreased after ampelopsin treatment, suggesting that ampelopsin may inhibit mitochondrial function in resistant cells via NF-κB (Figure 4C). Indeed, ampelopsin treatment significantly inhibited NF-κB signaling in MDA-MB-231/IR cells, as indicated by the reduced levels of p-IκBα/IκBα and p-NF-κB p65/NF-κB p65 (Figure 5B). TNF-α, a key pro-inflammatory cytokine, enhances the phosphorylation of IKK and regulates the phosphorylation of p65 on serine 536 [58]. In addition, overproduction of TNF-α is correlated with the development of multiple diseases, including cancer. Therefore, recent studies have attempted to develop phytochemicals to suppress TNF-α signaling [59]. Pretreatment with ampelopsin followed by exposure to TNF-α resulted in suppression of TNF-α-stimulated phosphorylation of IκBα and NF-κB p65 (Figure 5C), indicating that ampelopsin has potential for suppression of the TNF-α/NF-κB signaling in MDA-MB-231/IR cells. Further studies are required to access the mechanism underlying how NF-κB signaling influences stemness properties and mitochondrial function in breast CSCs.

## 4. Materials and Methods

### 4.1. Cell Culture

MDA-MB-231/IR cells were established from the parental MDA-MB-231 cells (ATCC, Rockville, MD, USA) and characterized as described previously [6], and then were cultured in DMEM (Gibco, Carlsbad, CA, USA) supplemented with 10% heat-inactivated fetal bovine serum (FBS, Gibco) and 1% antibiotic-antimycotic reagents (Gibco). Fibroblast cells were received from Professor Moonjae Cho and cultured in DMEM. MCF-10A cells were kept in accordance with ATCC recommendations. Medium was changed every 2–3 days. All cell lines were incubated at 37 °C under 5% CO_2_ atmosphere.

### 4.2. Cell Viability Assay

The MTT cell viability assay was conducted as previous described [60]. Briefly, cells (2 × 10^4^ cells/mL) were seeded into 96-well plates exposed to different doses of ampelopsin (TCI, Tokyo, Japan). After incubation of 12 h, 24, or 48 h, cells were exposed with 100 μL MTT reagent (1 mg/mL) for 2–3 h at 37 °C. Then, 150 μL of dimethyl sulfoxide (DMSO) was added to dissolve the formazan crystals. Following completion of the MTT assay, the absorbance was recorded by microplate reader (Tecan Group, Ltd., Salzburg, Austria) at 570 nm.

### 4.3. Wound Healing Assay

Cells (2 × 10^5^ cells/mL) were seeded into six-well plates. When cell density reached 95% confluence, a scratch wound was made in each well using a 200 µL sterile pipette tip, and then it was washed with phosphate-buffered saline (PBS). The cells were then treated with or without ampelopsin (dose 12.5 µM), followed by incubation for 24 h. Scratch in each well was examined under the light of a phase-contrast microscope.

### 4.4. Cell Invasion Assay

The cell invasion assay was conducted on 24-well Transwell plates (Corning, Cambridge, MA, USA) as described previously [60]. The upper chambers were coated with 1% Matrigel. After the gel had solidified, each well was loaded with 2 × 10^5^ cells in media-free FBS, with or without ampelopsin (dose 12.5 µM). The lower chamber was loaded with DMEM containing 10% FBS. The migratory cells were fixed with 4% paraformaldehyde. Further, 2% crystal violet was used to stain migrated cells. The stain cells were then observed under a light of a phase-contrast microscope.

### 4.5. Flow Cytometry Assay for CD44^+^/CD24^−/low^ Population

Cells (1 × 10^5^ cells/mL) were seeded into 60 mm dishes and then treated with different doses of ampelopsin (0, 25, 50, and 100 μM) for 24 h. After incubation, cells were suspended in immunofluorescence staining buffer comprising phytoerythrin-conjugated anti-human CD24 antibody and fluorescein isothiocyanate-conjugated anti-human CD44 antibody (BD Pharmingen, San Diego, CA, USA). The CD44^+^/CD24^−/low^ population was isolated by fluorescence-activated cell sorting (FACSCalibur, Becton Dickinson, Franklin Lakes, NJ, USA) at the Bio-Health Materials Core-Facility in Jeju National University.

### 4.6. ALDH Assay

The activity of ALDH enzyme was measured using an Aldefluor assay kit purchased from Stemcell Technologies as described previously [60]. Briefly, cells (1 × 10^5^ cells/mL) were seeded in 60 mm dishes and exposed for 24 h with ampelopsin at a non-toxic concentration (12.5 μM). Following incubation for 24 h, cells were added with Aldefluor assay kit according to the instructions of the manufacturer. FACSCalibur flow cytometer was used to identify ALDH-positive cells. The ALDH inhibitor, 4-diethylaminobenzaldehyde (DEAB), was added as a negative control.

### 4.7. Mammosphere Formation Assay

Cells (2 × 10^4^ cells/mL) were seeded in ultralow attachment dishes. The cells were grown in complete MammoCult Human Medium purchased from Stemcell Technologies and then treated with different concentration of ampelopsin (0, 25, 50, and 100 µM). After incubation for 10 days, mammospheres (size > 60 μm) were observed under the light of a phase-contrast microscope.

### 4.8. Colony Formation Assay

Cells (400 cells/mL) were seeded onto 60 mm plates and then incubated with different concentrations of ampelopsin (0, 3.125, 6.25, 12.5, 25, 50, 100, and 200 mM) for 10 days. The cells were then washed twice with PBS and fixed with 4% paraformaldehyde, followed by staining with 2% crystal violet for 30 min. The ImageJ software (NIH, Bethesda, MD, USA) was used to count cell colonies.

### 4.9. Annexin V/propidium Iodide Staining

Cells (1 × 10^5^ cells/mL) were seeded onto 60 mm plates and treated with ampelopsin at different doses (0, 25, 50, and 100 μM). The cells were then washed with PBS and the apoptotic population was stained using an Annexin V-FITC Apoptosis Detection Kit (BD Pharmingen) for 15 min at 37 °C under a dark cover. FACSCalibur flow cytometer was used to identify apoptotic cells.

### 4.10. Real-Time Polymerase Chain Reaction (PCR)

Cells (1 × 10^5^ cells/mL) were seeded in cell culture plates and then treated with ampelopsin (0, 50, and 100 µM) for 24 h. TRIzol reagent (Invitrogen, Carlsbad, CA, USA) was then used to extract total RNA. After reverse transcription of extracted total RNA, TOPreal™ qPCR 2× PreMIX kit (Enzynomics, Daejeon, South Korea) was used to conduct real-time PCR. The primers used are listed in Table S1. The 2^−ΔΔCt^ method described by Livak and Schmittgen was used to evaluate the gene expression [61].

### 4.11. Western Blotting Analysis

Western blotting was performed as previously described [62]. Briefly, cell density at 3 × 10^5^ was prepared on each 100 mm dish and cells were incubated in different doses of ampelopsin (0, 25, 50, and 100 μM) for 24 h. Then, radioimmunoprecipitation assay buffer (RIPA buffer) was used to extract cell lysates. Then, protein concentration in each sample was measured using a bicinchoninic acid (BCA) protein assay kit (Thermo Fisher Scientific, Waltham, MA, USA). After loading aliquots of 20–40 µg of protein, lysate was separated by sodium dodecyl sulfate-polyacrylamide gel electrophoresis (SDS-PAGE). The primary antibodies were bought from Cell Signaling Technology (Beverly, MA, USA), except the mouse anti-E-cadherin antibody (BD Transduction Laboratories, San Jose, CA, USA). The primary antibodies were diluted at 1:1000. The anti-GAPDH primary antibody was diluted at 1:7000. The anti-rabbit and anti-mouse immunoglobulin G (IgG) secondary antibodies (Vector Laboratories, Burlingame, CA, USA) were diluted at 1:5000 for 40–60 min at room temperature. The bands were detected using a BS ECL Plus kit (Biosesang, Seongnam, South Korea).

### 4.12. XF Seahorse Analysis

The OCR was accessed by a Seahorse XF cell mitochondria stress test kit from XF24 Extracellular Flux Analyzer (Seahorse Bioscience, Agilent Technologies, CA, USA). Cells (10^4^ cells/well) were seeded in Seahorse XF24 24-well plates. The next day, cells were treated with ampelopsin (0, 50, and 100 µM) dissolved in DMEM containing 10% FBS. After incubation for 24 h, the medium was replaced with warmed Seahorse medium (XF Assay Base Medium, pH 7.4) supplemented with 10 mM glucose, 1 mM sodium pyruvate, and 2 mM l-glutamine, and the cells were incubated for 1 h at 37 °C in a CO_2_-free incubator. The cells were then sequentially treated with oligomycin (2 μM), carbonyl cyanide-*p* trifluoromethoxyphenylhydrazone (1 μM), and rotenone (1 μM)/antimycin A (2 μM). The OCR was measured in pmol/min.

### 4.13. Statistical Analysis

The data were analyzed by GraphPad Prism 8.0 software. Data are displayed as means ± standard deviations of three independent experiments. Differences between treatments were examined by Student’s *t*-test. In all analyses, *p* < 0.05 was considered as statistical significance.

## 5. Conclusions

Ampelopsin is one of the major flavonoids found in the members of the species *Ampelopsis*. To the best of our knowledge, this study is the first to demonstrate that ampelopsin inhibited the proliferation and induced apoptosis of chemo- and radio-resistant TNBC cells. Notably, ampelopsin impaired the stem-like characteristics and mitochondrial function of resistant MDA-MB-231/IR cells. Ampelopsin suppressed NF-κB signaling in MDA-MB-231/IR cells, suggesting that it is a promising novel agent for CSC targeting in TNBC patients.

## Data Availability

All data is available within the article and Supplementary Materials.

## Supplementary Materials

The following are available online at https://www.mdpi.com/article/10.3390/ph14080794/s1: Figure S1: Effects of flavonoid compounds on viability of MDA-MB-231 and MDA-MB-231/IR cells; Figure S2: Expression of AMPK and mTOR in MDA-MB-231 and MDA-MB-231/IR cells determined by Western blotting analysis; Figure S3: Original films of Figure 1D; Figure S4: Original films of Figure 2D; Figure S5: Original films of Figure 3C; Figure S6: Original films of Figure 4A; Figure S7: Original films of Figure 5A; Figure S8: Original films of Figure 5B; Figure S9: Original films of Figure 5C; Figure S10: Original films of Figure S2. Table S1: Primer sequences for OXPHOS-related genes.

## Abbreviations

Akt	Protein kinase B
ALDH	Aldehyde dehydrogenases
AMPK	AMP-activated protein kinase
BCSCs	Breast cancer stem cells
CSCs	Cancer stem cells
DEAB	Dimethylaminobenzaldehyde
EMT	Epithelial–mesenchymal transition
ER	Estrogen receptor
FACS	Fluorescence-activated cell sorting
FBS	Fetal bovine serum
HER2	Human epidermal growth factor receptor 2
IC_50_	Inhibitory concentration of 50
IgG	Immunoglobulin G
IκB	I-kappa-B
IKK	IκB kinase
IR	Irradiation
KLF4	Krüppel-like factor 4
MMP	Matrix metalloproteinase
MRP1	Multidrug resistance-associated protein 1
mTOR	Mammalian target of rapamycin
MTT	3-(4,5-dimethylthiazol-2-yl)-2,5-diphenyl-tetrazolium bromide
NF-κB	Nuclear factor-κB
OCR	Oxygen consumption rate
OXPHOS	Oxidative phosphorylation
PCR	Polymerase chain reaction
PR	Progesterone receptor
TNBC	Triple negative breast cancer
TNF-α	Tumor necrosis factor α
