# Supplementary material for: Ampelopsin Suppresses Stem Cell Properties Accompanied by Attenuation of Oxidative Phosphorylation in Chemo- and Radio-Resistant MDA-MB-231 Breast Cancer Cells
Source: Pharmaceuticals (Basel). 2021 Aug 12;14(8):794. doi: 10.3390/ph14080794 (PMC8400665; doi:10.3390/ph14080794)
Supplement: Supplementary file 1 [file pharmaceuticals-14-00794-s001.zip › pharmaceuticals-1273522-supplementary.pdf]

Supplementary Materials

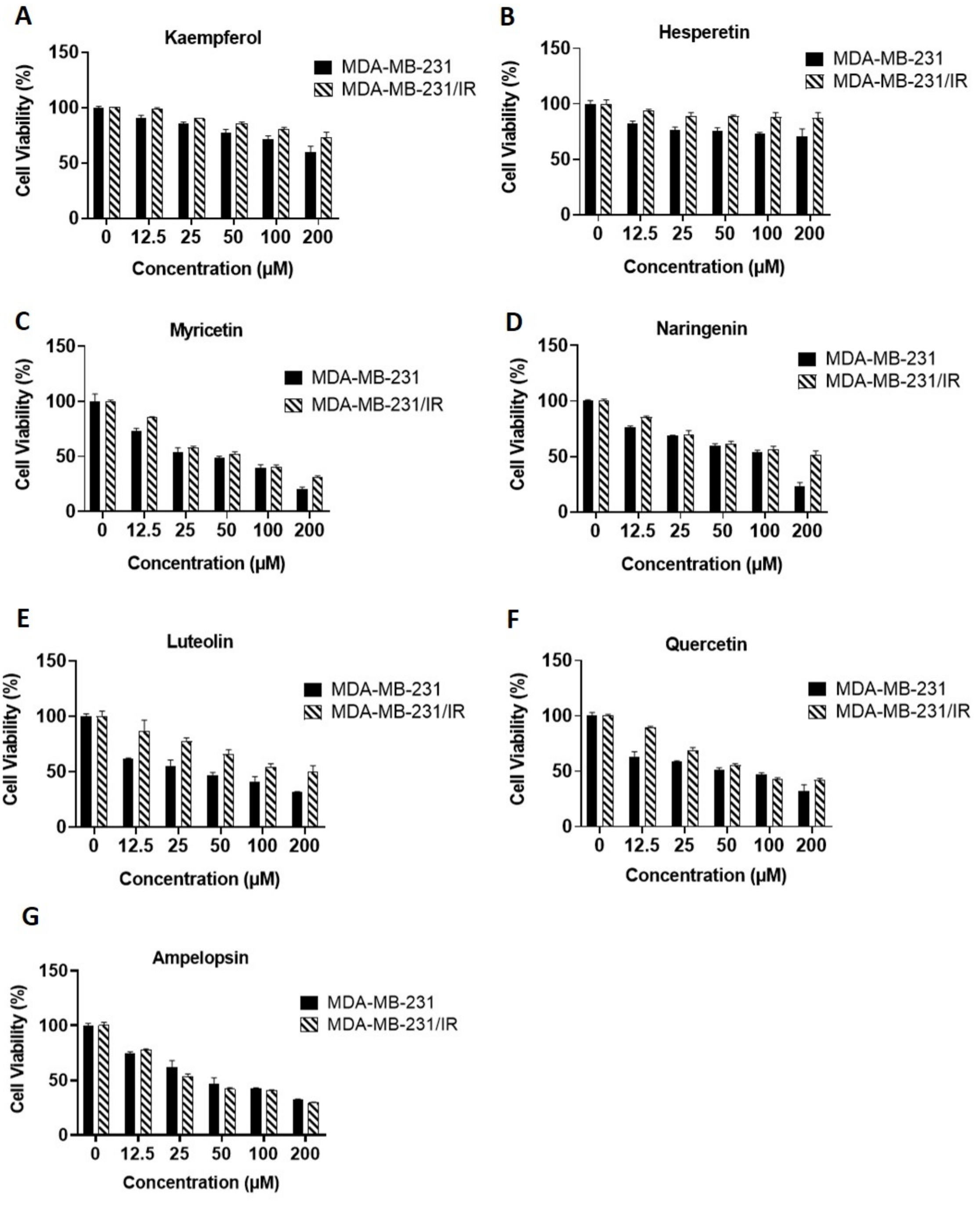

Figure S1: Effects of flavonoid compounds on viability of MDA-MB-231 and MDA-MB-231/IR cells. Effects on cell viability of incubation for 24 h with (A) kaempferol, (B) hesperetin, (C) myricetin , (D) naringenin, (E) luteolin, (F) quercetin and (G) ampelopsin were evaluated by MTT assay. Only ampelopsin had higher cytotoxicity in MDA-MB-231/IR cells than in MDA-MB-231 cells. Data are shown as means ± standard deviations of three independent experiments.

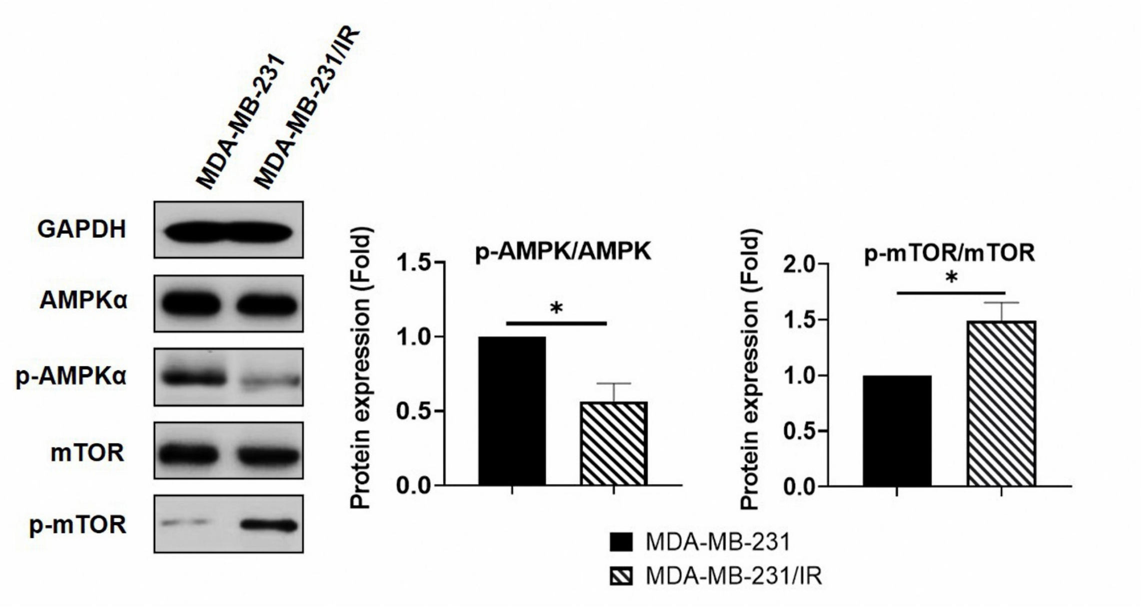

Figure S2: Expression of AMPK and mTOR in MDA-MB-231 and MDA-MB-231/IR cells determined by Western blotting analysis. MDA-MB-231/IR cells showed reduced AMPK activation and increased mTOR phosphorylation, compared with MDA-MB-231 cells. GAPDH was used as a control. Data are shown as means ± standard deviations of three independent experiments. * $p < 0.05$ vs. control.

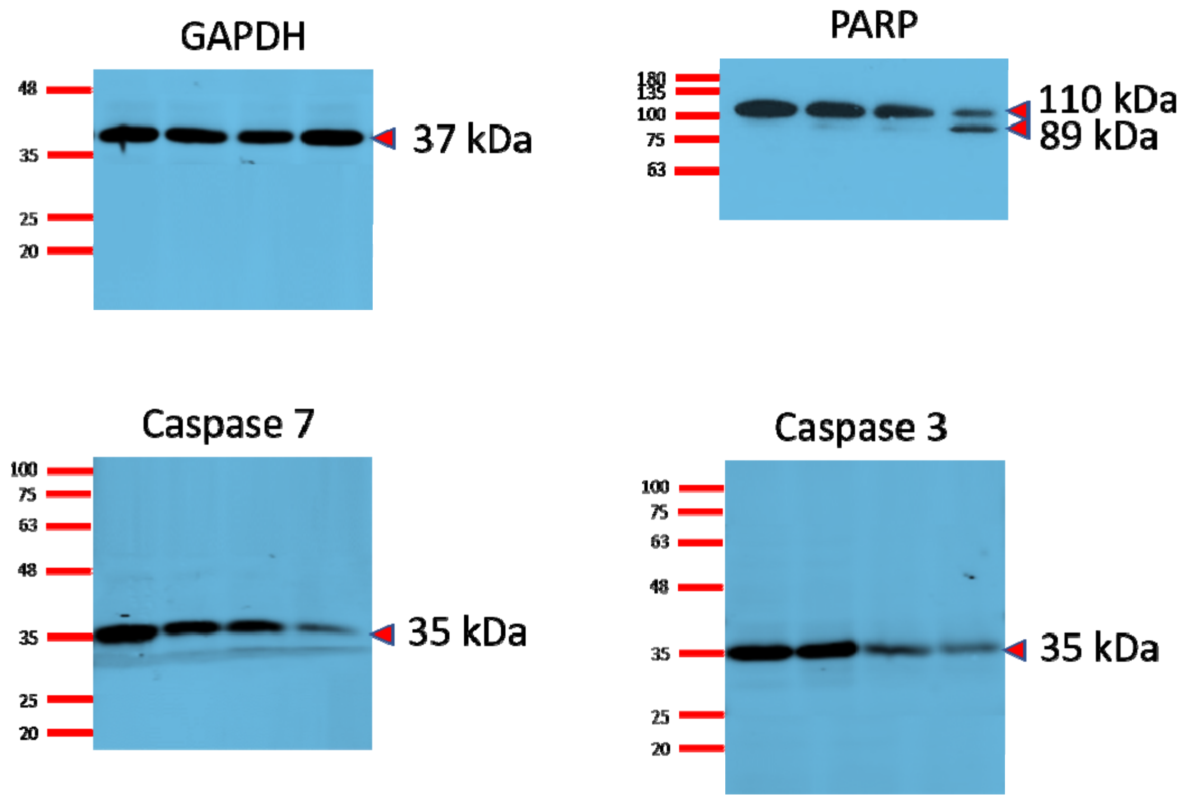

Figure S3: Original films of Figure 1D.

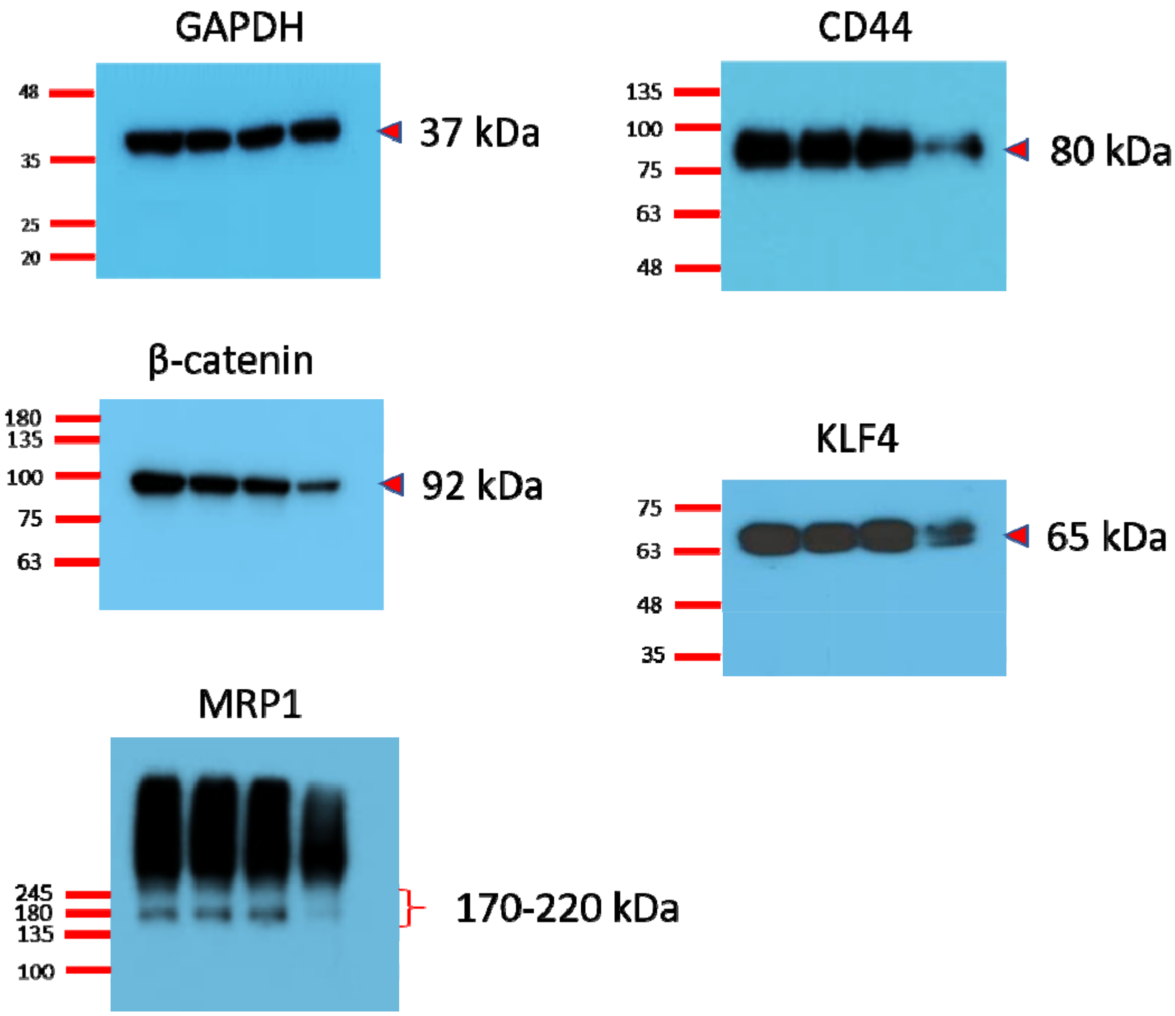

Figure S4: Original films of Figure 2D.

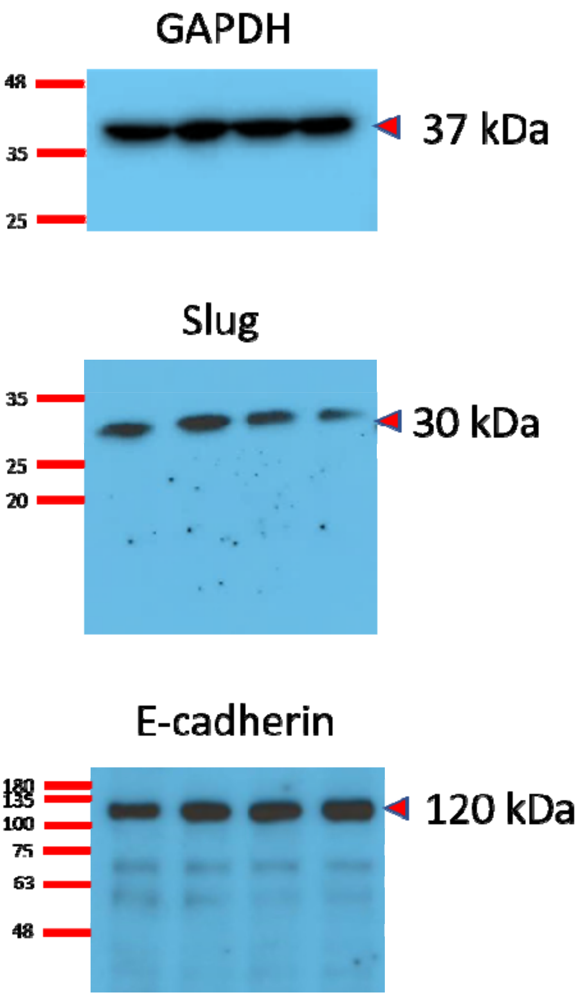

Figure S5: Original films of Figure 3C.

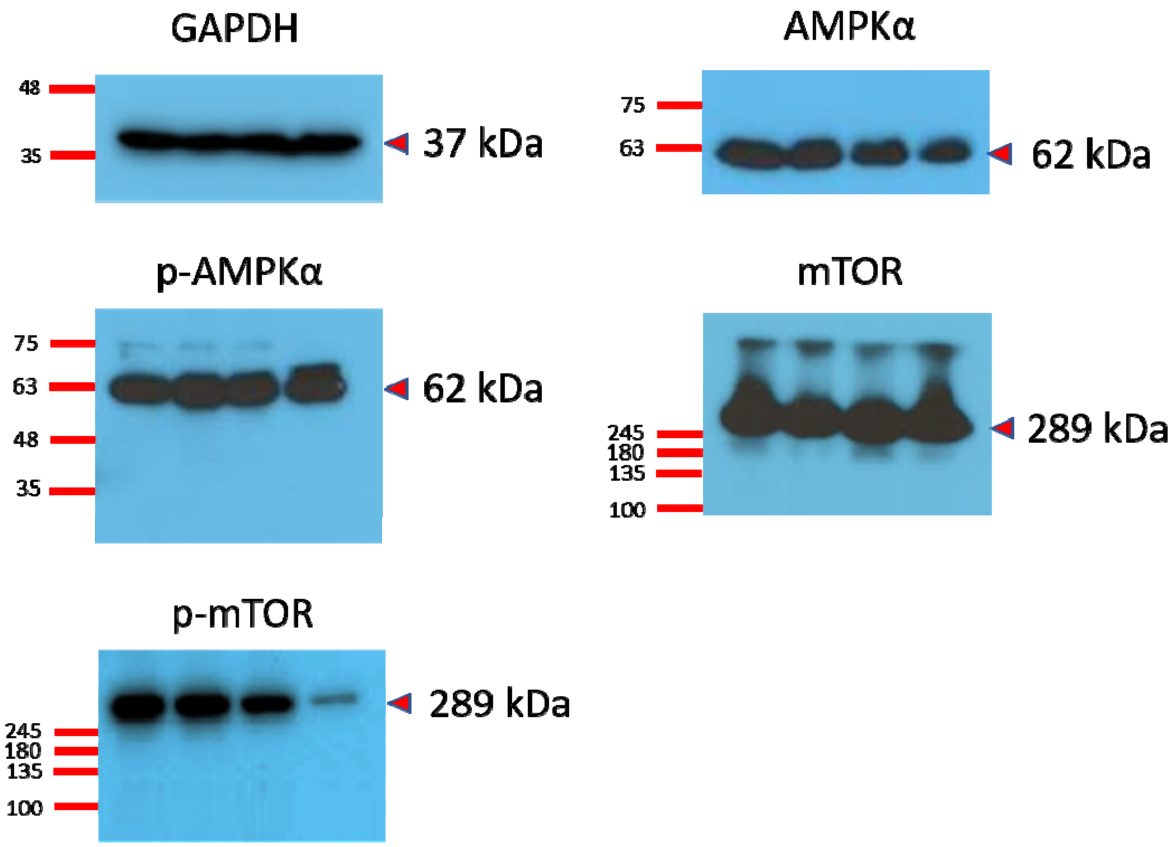

Figure S6: Original films of Figure 4A.

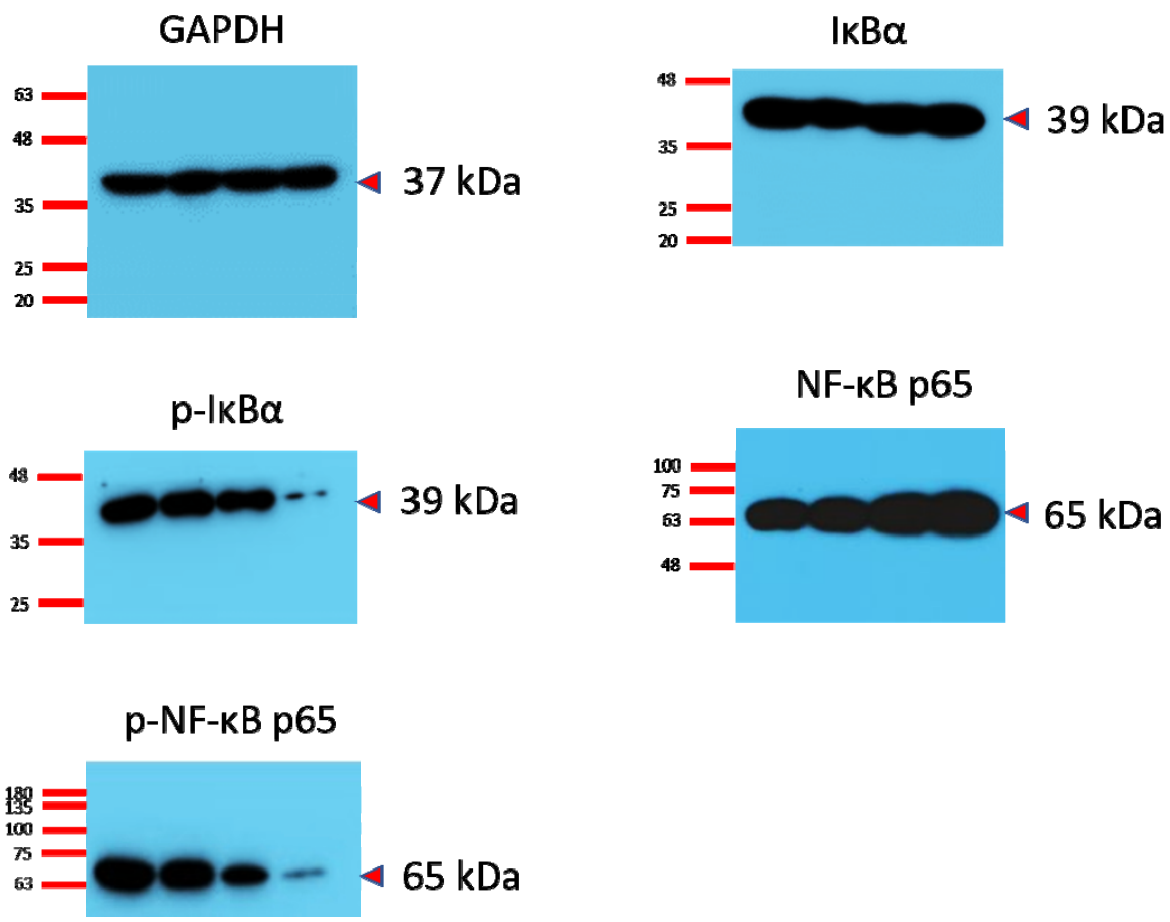

Figure S7: Original films of Figure 5A.

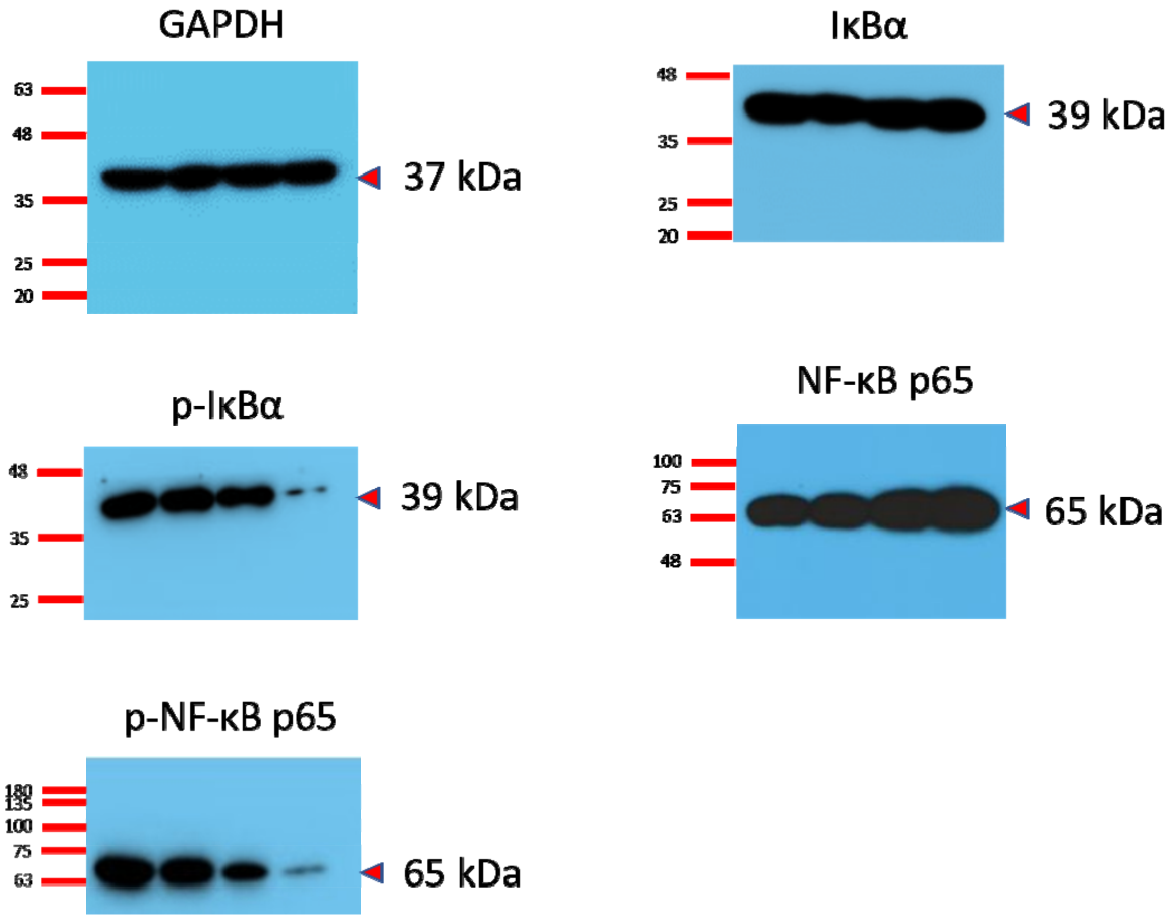

Figure S8: Original films of Figure 5B.

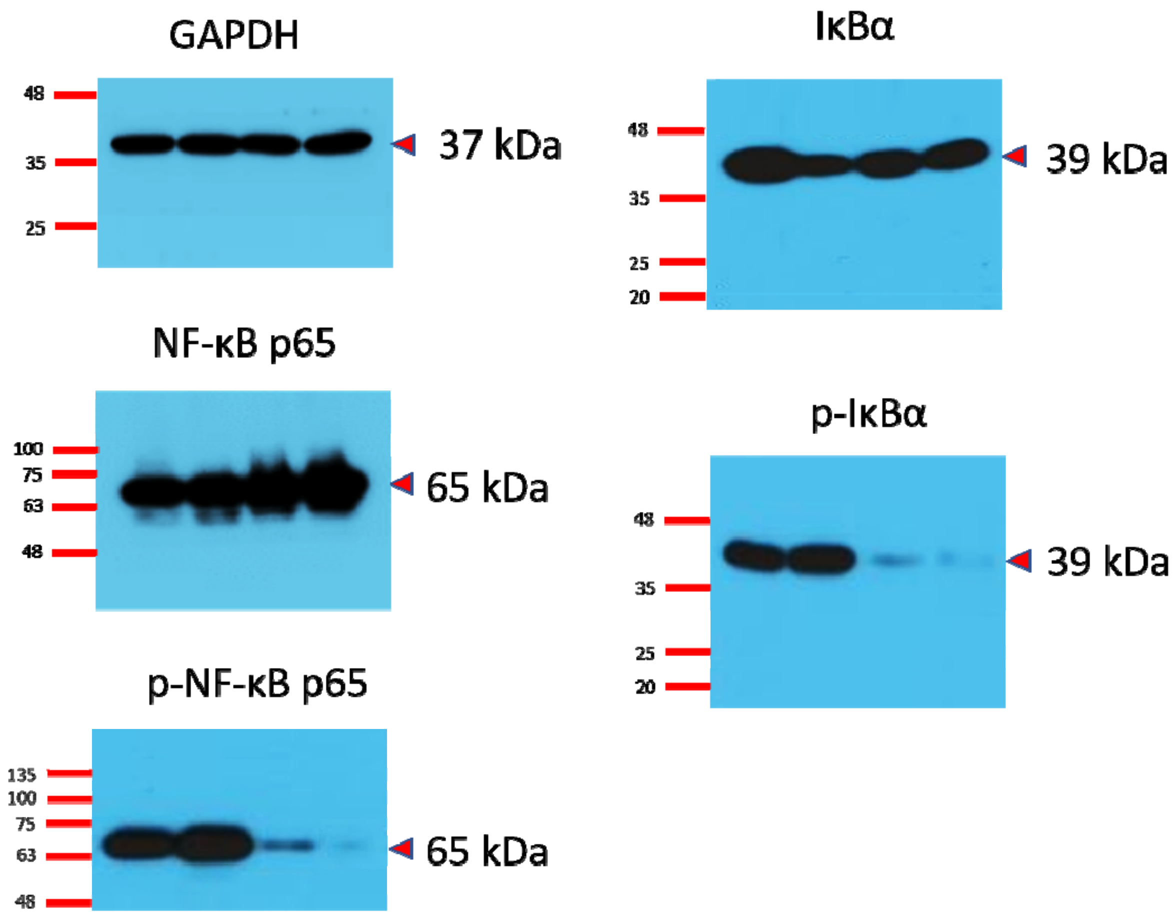

Figure S9: Original films of Figure 5C.

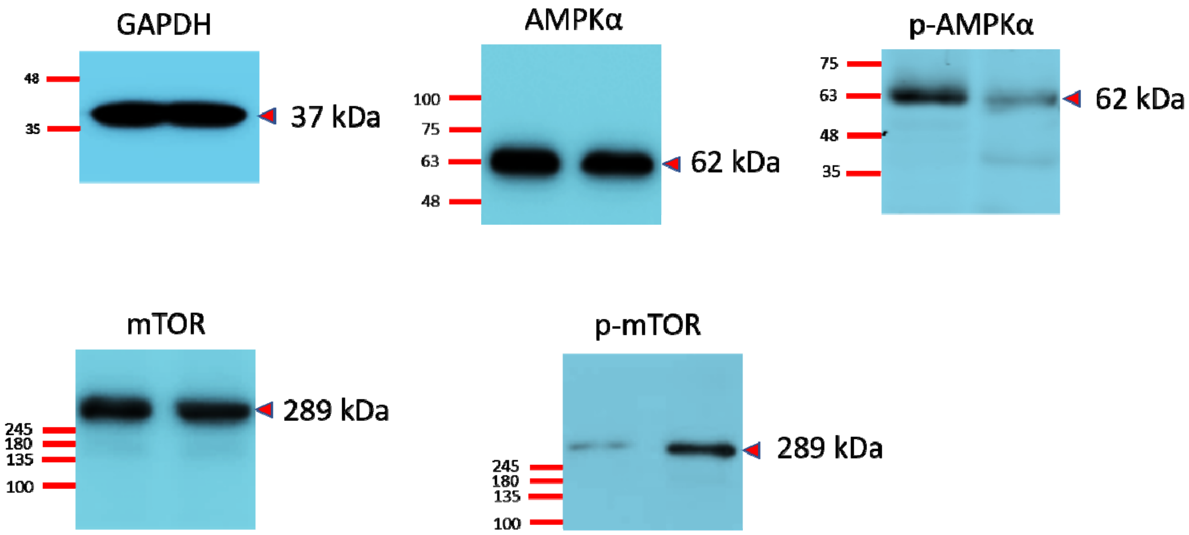

Figure S10: Original films of Figure S2.

Table S1: Primer sequences for OXPHOS-related genes.

Gene	Primer sequences
NDUFA10	5′-CACCTGCGATTACTGGTTCAG-3′<br>5′-GCAGCTCTCTGAACTGATGTA-3′
SDHB	5′-AGCCTTATCTGAAGAAGAAGG-3′<br>5′-TACTTGTCTCCGTTCCACCAG-3′
COX5B	5′-CAGAAGGGACTGGACCCATA-3′<br>5′-TTCACAGATGCAGCCCACTA-3′
COX4I1	5′-ACGAGCTCATGAAAGTGTTGTG-3′<br>5′-AATGCGATACAACTCGACTTTCTC-3′
ATP5G3	5′-GGATTTGCCTTGTCTGAAGC-3′<br>5′-CGTACATTCCCATGACACCA-3′
SCO2	5′-TGGGTGCTGATGTACTTTGGC-3′<br>5′-ACAGTCTTGGGTGGAAGTCCTG-3′
CYC1	5′-TAGAGTTTGACGATGGCACCC-3′<br>5′-CGTTTTCGATGGTCGTGCTC-3′
UQCRC1	5′-ACGGTGGGAGTGTGGATTGAC-3′<br>5′-CATTGCCAGGCCGATTCTTTG-3′
ATP5B	5′-GGCACAATGCAGGAAAGG-3′<br>5′-TCGGCAGGCACATAGATAGCC-3′
PGC1-α	5′-TTCCACCAAGAGCAAGTAT-3′<br>5′-CGCTGTCCCATGAGGTATT-3′
